# MMP25 Regulates Immune Infiltration Level and Survival Outcome in Head and Neck Cancer Patients

**DOI:** 10.3389/fonc.2020.01088

**Published:** 2020-07-29

**Authors:** Yujie Liang, Chenyu Guan, Kan Li, Guangsen Zheng, Tao Wang, Sien Zhang, Guiqing Liao

**Affiliations:** ^1^Department of Oral and Maxillofacial Surgery, Guanghua School of Stomatology, Hospital of Stomatology, Sun Yat-sen University, Guangzhou, China; ^2^Guangdong Province Key Laboratory of Stomatology, Sun Yat-sen University, Guangzhou, China

**Keywords:** MMP25, head and neck cancer, immune infiltration level, TCGA, bioinformatics

## Abstract

**Background:** MMP25 is a critical gene of matrix metalloproteinases (MMPs). However, the molecular mechanism of MMP25 in head and neck cancer pathogenesis remains unclear.

**Methods:** MMP25 expression was analyzed using The Cancer Genome Atlas (TCGA) database, and its influence on clinical prognosis was performed using Kaplan–Meier and Cox regression analyses. The correlation between MMP25 and immune infiltration was investigated by CIBERSORT, TIMER, and ESTIMATE. In addition, the relationship between MMP25 expression and molecular mechanisms was analyzed by gene set enrichment analysis (GSEA), gene ontology (GO), and weighted gene co-expression network analysis (WGCNA).

**Results:** MMP25 expression level correlated with prognosis and immune infiltrating levels, especially activated CD4^+^ memory T cells, in head and neck cancer. Moreover, MMP25 expression potentially mediated genes, such as IRF8, IKZF1, and DOCK2, and tumor-associated pathways, including p53 signaling, PI3K/AKT/mTOR signaling, and JAK/STAT signaling pathway.

**Conclusions:** These findings suggested that MMP25 plays a critical role in the prognosis and immune infiltration level of head and neck cancer. In addition, MMP25 expression significantly correlated with the regulation of various oncogenes and tumor-related pathways.

## Introduction

Head and neck cancer was the seventh most common cancer worldwide (1), originating from multiple anatomical structures:the oral cavity, sinonasal cavity, pharynx, and larynx (2). Advanced disease carries a high risk of local recurrence and metastasis, with poor prognosis (3, 4). Immune checkpoint inhibitors have been considered as a promising strategy for the treatment of head and neck cancer (5). Clinical trials have shown a clear prognostic advantage in head and neck cancer patients treated with immunotherapy (6). However, an increasing number of studies found that some types of head and neck cancer were insensitive to current immunotherapies (7). Moreover, a recent study has found that tumor-infiltrating immune cells, such as tumor-associated macrophages (TAMs) and regulatory T cells (Tregs) (8), impaired the prognosis and efficacy of immunotherapy in head and neck cancer.

MMPs are calcium-dependent zinc-containing endopeptidases which can degrade the extracellular matrix of tumor tissues and regulate tumor immune environment, and promote cancer invasion and metastasis (9, 10). MMP25 is a member of the matrix metalloproteinase family that is able to degrade collagens, gelatin, and fibrin (11). However, the underlying functions of MMP25 in the head and neck cancer still require further investigation. In this study, we comprehensively analyzed the mechanism by which MMP25 expression influences the prognosis of head and neck cancer patients. Our results showed that MMP25 high expression was related to head and neck cancer patients' better outcome, and that was regulated by oncogenes and cancer-associated pathways. In addition, we found that MMP25 had a significant effect on immune infiltration level in head and neck cancer. The findings shed light on the role of MMP25 in head and neck cancer by providing a potential correlation and a precise mechanism between MMP25 and tumor immune microenvironment.

## Materials and Methods

### Data Source

Expression data and corresponding clinical information of head and neck cancer patients were downloaded from The Cancer Genome Atlas (TCGA) (Tumor samples: *n* = 502, Normal samples: *n* = 44).

### Transcriptional Expression of Matrix Metalloproteinases

MMP1, MMP2, MMP3, MMP7, MMP8, MMP9, MMP10, MMP11, MMP12, MMP13, MMP14, MMP15, MMP16, MMP17, MMP19, MMP20, MMP21, MMP23B, MMP24, MMP24OS, MMP25, MMP26, MMP27, and MMP28 were formed with the FPKM of each mRNA expression of samples. Then, comparisons between tumor tissues and normal tissues were analyzed.

### Prognostic Analysis of Head and Neck Patients

The clinical outcome of head and neck patients was determined using the Kaplan–Meier method, the log-rank test, and univariate Cox proportional hazards regression. The samples were stratified as high or low expression around the quartile of each MMPs' expression.

### Identification of Differentially Expressed Genes

Differentially expressed genes (DEGs) were identified in head and neck cancer tissues by comparison with MMP25 high and low expression groups and using the “edgeR” package in R (R version 3.5.2). |Fold Change| > 2 and adjusted *p* < 0.05 were set as the statistical threshold value of DEGs. The heatmap, volcano plot with clustering for the significantly differentially expressed mRNAs in head and neck cancer between MMP25 high and low expression groups, was generated with the “ggplot2” package in R. GO analysis was constructed by “enrichplot” in R.

### Gene Set Enrichment Analysis (GSEA)

The molecular mechanisms underlying the association between MMP25 expression were explored with GSEA. The number of permutations was set at 1,000, and the *p* < 0.05 and a false discovery rate (FDR) <0.25 were considered statistically significant. Multiple GSEA plots were produced by “plyr,” “ggplot2,” and “grid” packages in R.

### The Clinical Features Correlated With MMP25 Expression Level

Clinical information from head and neck cancer patients, including age, gender, tumor grade, and clinical stage, was downloaded from TCGA. Samples were divided into two groups and according to the quartile of MMP25 expression level. Multivariate Cox regression and nomogram were used to analyze the role of MMP25 in the head and neck cancer patients' clinical features.

### Immune Landscape Related to MMP25 Expression Level

The samples were analyzed by CIBERSORT to define 22 immune cell subtypes (12). Immune scores were calculated by ESTIMATE algorithm of immune cells (13). The correlations between MMP25 expression levels and immune infiltration level was estimated by TIMER, which is a comprehensive resource for analysis of immune infiltrates of gene expression profiles (14).

### Weighted Gene Co-expression Network Analysis

The weighted gene co-expression network analysis (WGCNA) (15, 16) package was used to identify key modules associated with prognosis and clinical stage based on MMP25 expression levels. The module is a cluster of closely interconnected genes, based on the dendrogram height. The module eigengenes of clinical features were hierarchically clustered into different color modules.

### Statistical Analysis

The analyses were carried out using “R” software (version 3.5.3), GraphPad Prism 8, and IBM SPSS Statistics 19; *p* < 0.05 was considered statistically significant. ^***^p < 0.001, ^**^p < 0.01 ^*^p < 0.05.

## Results

### Expression of Matrix Metalloproteinases Family in Head and Neck Cancer

**Figure 1:**
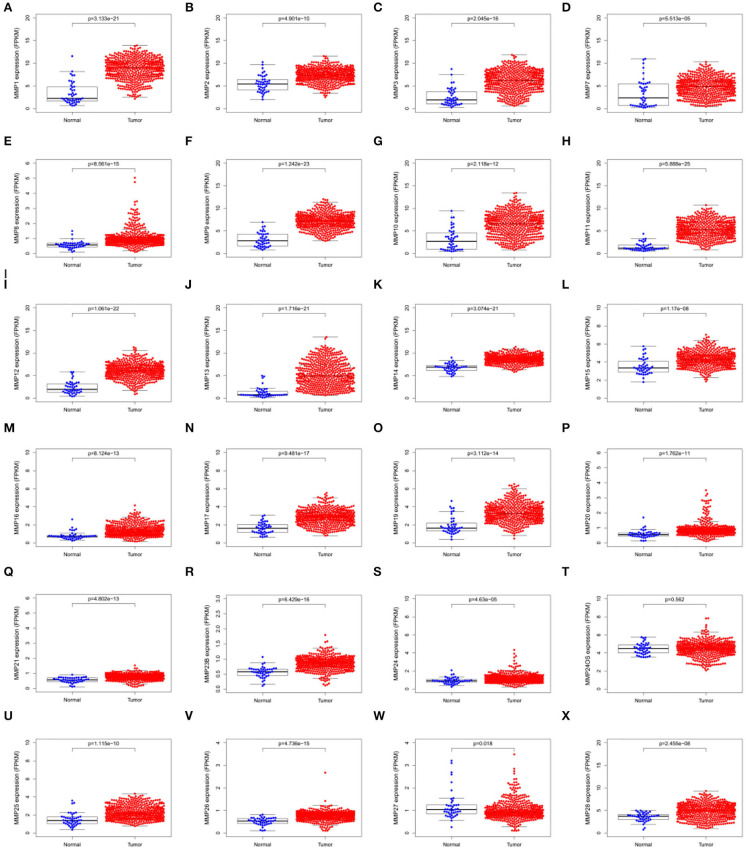
Scatter plots showing the expression levels of matrix metalloproteinases family genes of normal tissues (blue) and tumor tissues (red) in head and neck cancer. **(A)** MMP1; **(B)** MMP2; **(C)** MMP3; **(D)**MMP7; **(E)** MMP8; **(F)** MMP9; **(G)**MMP10; **(H)** MMP11; **(I)** MMP12; **(J)** MMP13; **(K)**MMP14; **(L)**MMP15; **(M)**MMP16; **(N)** MMP17; **(O)** MMP19; **(P)** MMP20; **(Q)** MMP21; **(R)**MMP23B; **(S)** MMP24; **(T)** MMP24OS; **(U)** MMP25; **(V)** MMP26; **(W)** MMP27; **(X)** MMP28.

The expression levels of matrix metalloproteinases family genes are shown in Figure 1. Except for MMP24OS, the transcription level of other MMP family genes, including MMP1, MMP2, MMP3, MMP7, MMP8, MMP9, MMP10, MMP11, MMP12, MMP13, MMP14, MMP15, MMP16, MMP17, MMP19, MMP20, MMP21, MMP23B, MMP24, MMP25, MMP26, MMP27, and MMP28, in the tumor tissues was significantly higher than those in normal tissues.

### Analysis of the Prognosis Associated With MMPs Expression Levels in TCGA Cohort of Head and Neck Cancer

**Figure 2:**
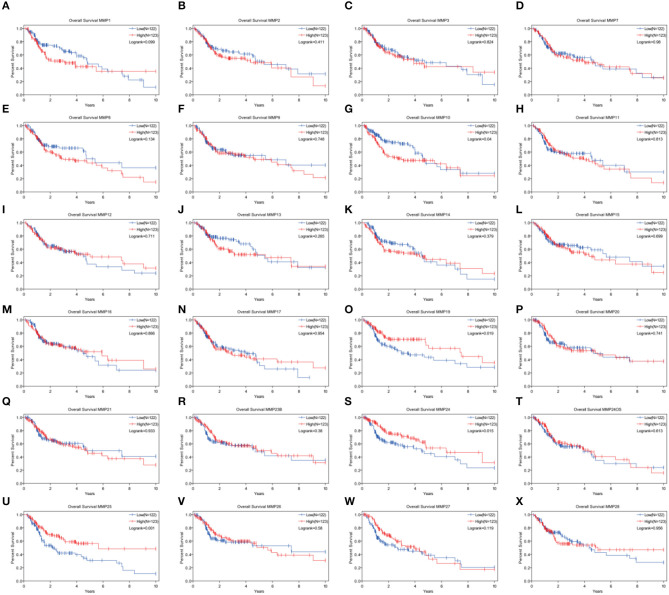
Analysis of the role of MMPs genes in the overall survival in head and neck cancer patients by Kaplan–Meier method and the log-rank test. **(A)** MMP1; **(B)** MMP2; **(C)** MMP3; **(D)** MMP7; **(E)** MMP8; **(F)** MMP9; **(G)**MMP10; **(H)** MMP11; **(I)** MMP12; **(J)** MMP13; **(K)** MMP14; **(L)** MMP15; **(M)** MMP16; **(N)** MMP17; **(O)** MMP19; **(P)** MMP20; **(Q)** MMP21; **(R)** MMP23B; **(S)** MMP24; **(T)** MMP24OS; **(U)** MMP25; **(V)** MMP26; **(W)** MMP27; **(X)** MMP28.

Expression levels of MMP10, MMP19, MMP24, and MMP25, which were associated with prognosis in head and neck cancer, were identified using Kaplan–Meier method and log-rank test (Figure 2, Table 1). Univariate Cox regression analysis showed that there was no significant dereference between expression levels of MMP10 (*p* = 0.16), MMP19 (*p* = 0.06), and MMP24 (*p* = 0.30). Eventually, MMP25 was identified.

**Table 1:** Univariate Cox proportional hazards regression analysis of the role of matrix metalloproteinases (MMPs) genes of head and neck cancer (HNSC).

**Gene**	**HR[exp(coef)]**	**coef**	**95% CI lower**	**95% CI upper**	***p*-value**
MMP25	0.81	−0.21	−0.41	−0.01	0.04
MMP8	1.26	0.23	0.00	0.46	0.05
MMP19	0.88	−0.13	−0.27	0.01	0.06
MMP1	1.06	0.06	−0.01	0.12	0.08
MMP14	1.15	0.14	−0.02	0.29	0.09
MMP10	1.04	0.04	−0.02	0.10	0.16
MMP3	1.04	0.04	−0.02	0.10	0.20
MMP24	0.84	−0.17	−0.50	0.15	0.30
MMP13	1.03	0.03	−0.03	0.08	0.33
MMP7	1.02	0.02	−0.05	0.09	0.52
MMP15	1.05	0.05	−0.12	0.22	0.56
MMP27	0.91	−0.10	−0.45	0.25	0.58
MMP17	0.96	−0.05	−0.22	0.13	0.61
MMP16	0.95	−0.05	−0.27	0.18	0.68
MMP9	1.02	0.02	−0.07	0.10	0.69
MMP20	0.93	−0.07	−0.43	0.29	0.71
MMP12	1.02	0.02	−0.07	0.10	0.71
MMP2	1.02	0.02	−0.08	0.11	0.71
MMP21	1.14	0.13	−0.60	0.85	0.73
MMP11	0.99	−0.01	−0.08	0.07	0.88
MMP23B	1.04	0.04	−0.60	0.69	0.89
MMP26	1.04	0.04	−0.71	0.78	0.92
MMP28	1.00	0.00	−0.09	0.09	0.98
MMP24OS	1.00	0.00	−0.18	0.18	0.98

### Analysis of the Clinical Features Associated With MMP25 Expression Levels in TCGA Cohort of Head and Neck Cancer

**Figure 3:**
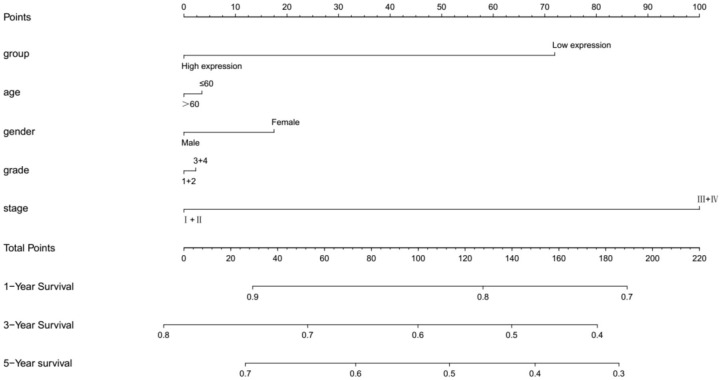
Nomogramfor predicting 1-, 3-, or 5-year survival in head and neck cancer patients. The top row shows the point value for each variable. Rows indicate the variables included in the nomogram. Each variable corresponds to a point value based on head and neck cancer characteristics. The sum of these values is located on the Total Points, and the line drawn downward to the survival axes is used to determine the likelihood of 1-, 3-, or 5-year survival.

In TCGA cohort, multivariate Cox regression model indicated that MMP25 expression was correlated with clinical stage with prognosis of head and neck cancer patients in terms of overall survival in the TCGA cohort (Table 2). In view of the prognostic value of MMP25 in head and neck cancer, we tried to construct the nomogram for predicting 1-, 3-, and 5-year survival. The result illustrated that clinical stages shared the largest contribution to overall survival, followed by MMP25 expression group (Figure 3).

**Table 2:** Multivariate Cox regression of clinical characteristics of HNSC patients in The Cancer Genome Atlas (TCGA) cohort.

**Variables**	**MMP25**		
	**High (*n* = 100)**	**Low (*n* = 101)**	***p-*value**
**Age**			0.91
≤ 60	46	46	
>60	54	55	
**Gender**			0.59
Male	64	74	
Female	36	27	
**Grade**			0.95
Grade (1+2)	70	82	
Grade (3+4)	30	19	
**Stage**			0.019
Stage (I+II)	32	13	
Stage (III+IV)	68	88	

### Analysis of the Differential Expressed Genes With MMP25 High and Low Expression Group of Head and Neck Cancer

**Figure 4:**
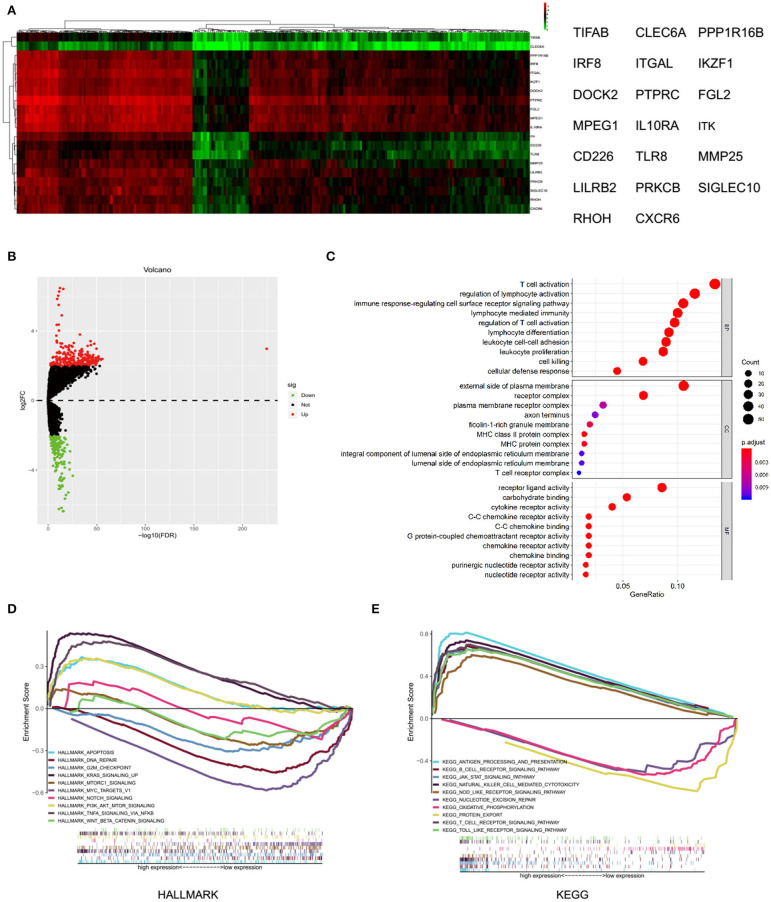
Differential expressed genes and gene set enrichment analysis (GSEA) with MMP25 high and low expression group. **(A)** Heatmap of differential expressed genes analysis by “edgeR” package in R. **(B)** Volcano plot of differential expressed genes with MMP25 high and low expression group. **(C)** GO analysis of differential expressed genes. **(D)** Hallmark enrichment analysis by GSEA. **(E)** KEGG enrichment analysis by GSEA.

A total of 433 differential expressed genes, including 239 upregulated and 194 downregulated DEGs, was screened with MMP25 high and low expression group (|logFC | > 2 and adjusted *p* < 0.05) (Figure 4B). The top 20 differential expressed genes were TIFAB, CLEC6A, PPP1R16B, IRF8, ITGAL, IKZF1, DOCK2, PTPRC, FGL2, MPEG1, IL10RA, ITK, CD226, TLR8, MMP25, LILRB2, PRKCB, SIGLEC10, RHOH, and CXCR6 (Figure 4A). The expression levels of these genes were positively correlated with the expression level of MMP25. The GO analysis showed that the differentially expressed genes were highly associated with T cell activation, external side of plasma membrane, and receptor ligand activity (Figure 4C). The significant pathways for these two groups were mainly enriched in the KRAS signaling pathway, MYC-targets, antigen processing and presentation, and protein export. In addition, tumor-associated pathways, such as apoptosis, PI3K/AKT/mTOR signaling pathway, JAK/STAT signaling pathway, and T cell receptor signaling pathway, were also highly enriched (Figures 4D,E).

### MMP25 Is Associated With mRNA Subtype Specific Immune Cell Infiltration Patterns in Head and Neck Cancer

**Figure 5:**
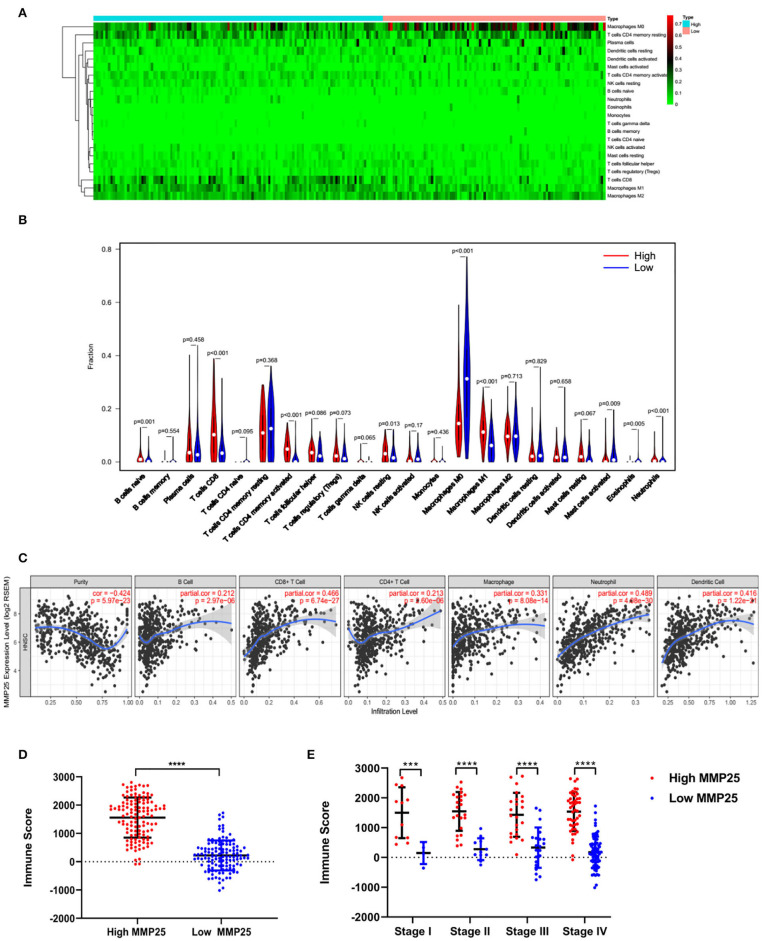
Immune cell infiltration patterns and scores between MMP25 high and low expression group. **(A)** Heatmap of immune infiltration level. **(B)** Violin plot of immune infiltration level between MMP25 high (red) and low (blue) expression group. **(C)** The correlation between the MMP25 expression level and immune infiltration level by TIMER database. **(D)** Difference of immune scores between two groups. **(E)** Difference of immune scores among clinical stages.

The CIBERSORT analysis indicated that the MMP25 high expression group had significantly higher B cells naïve, CD8^+^ T cells, activated CD4^+^ memory T cells, resting NK cells, M1 macrophage, and neutrophils, but lower M0 macrophage and eosinophil (Figures 5A,B). The TIMER database revealed that MMP25 expression was positively correlated with immune cells infiltration, including B cell, CD8^+^T cell, CD4^+^ T cell, and macrophage in head and neck cancers (Figure 5C). The graph also showed that there has been an increase of immune scores in the MMP25 high expression group of different clinical stages (Figures 5D,E). The Kaplan–Meier method analysis of CIBERSORT data suggested that the infiltration level of activated memory CD4^+^ T cells was significantly related to the survival outcome of head and neck cancer patients. The infiltration level of activated memory CD4^+^ T cells was positively correlated with the MMP25 expression level (Supplemental Figures 1A,B). These findings showed that a high infiltration level of activated memory CD4^+^ T cells played an important role in the better prognosis of cancer patients in the MMP25 high expression group.

### Identify Key Modules Associated With Head and Neck Cancer Patients' Survival Ratio and Clinical Stage Based on the Expression Levels of MMP25

**Figure 6:**
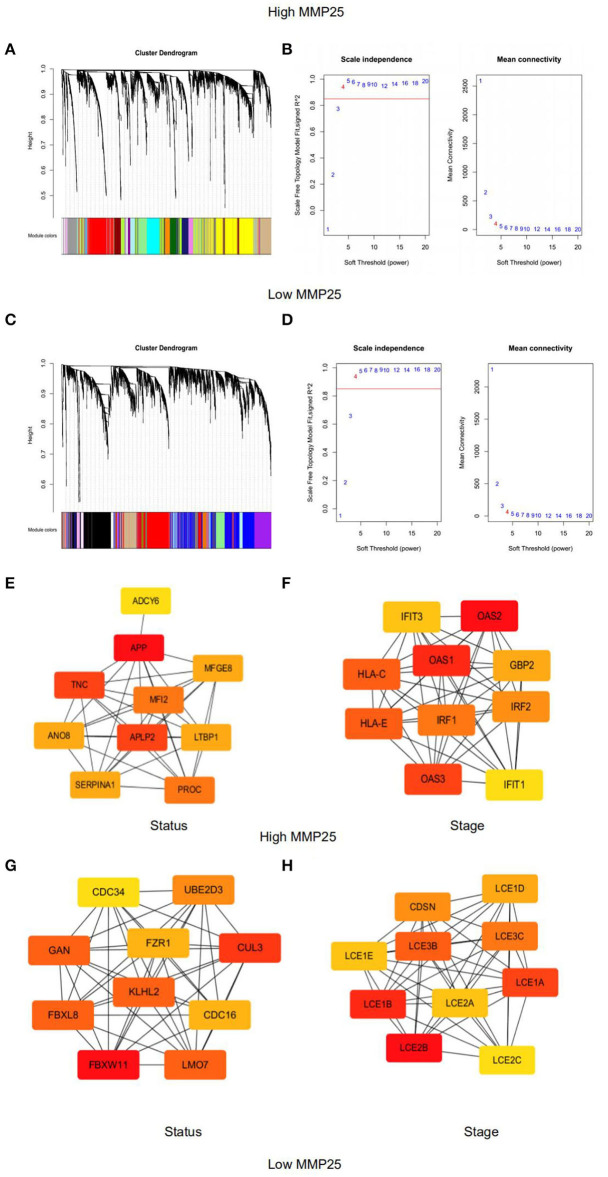
Weighted gene co-expression network analysis of module eigengenes of grade correlated with **(A)** Clustering dendrograms of genes in the MMP25 high expression group. **(B)** The scale-free topology model fit for various soft-thresholding powers (β) in MMP25 high expression group. **(C)** Clustering dendrograms of genes in the MMP25 low expression group. **(D)** The scale-free topology model fit for various soft-thresholding powers (β) in MMP25 low expression group. **(E)** Hub genes protein interaction network of the significant modules for patients' status in MMP25 high expression group. **(F)** Hub genes protein interaction network of the significant modules for patients' stages in MMP25 high expression group. **(G)** Hub genes protein interaction network of the significant modules for patients' status in MMP25 low expression group. **(H)** Hub genes protein interaction network of the significant modules for patients' stages in MMP25 low expression group.

Gene modules were analyzed using the WGCNA in the MMP25 high and low expression groups, respectively. In the high expression group, soft power 4 and the minimum module size cut-off 30 were chosen as the threshold to identify co-expressed gene modules (Figure 6A). Gene color modules that were related to the clinical stage were identified (Supplemental Figure 2). These significant gene color modules were further used for GO analysis to display the gene pathway enrichment, gene symbols, and their character in KEGG, as shown in Supplemental Tables 1, 2. The genes were related to many pathways in cancer, such as PI3K-Akt signaling pathway, p53 signaling pathway, Ras signaling pathway, MAPK signaling pathway, and TNF signaling pathway. In addition, genes were also enriched in cell cycle, DNA replication, and mismatch in cancer. Hub genes ADCY6, APP, TNC, MFGE8, MFI2, ANO8, APLP2, LTBP1, SERPINA1, and PROC were highly related to the survival outcome of patients. IFIT3, OAS2, OAS1, HLA-C, HLA-E, IRF1, GBP2, IRF2, OAS3, and IFIT1 were closely associated with the patients' clinical stages. However, in the low expression group, hub genes CDC34, UBE2D3, GAN, FZR1, CUL3, KLHL2, FBXL8, CDC16, FBXW11, and LMO7 were highly related to the survival outcome. CDSN, LCE1D, LCE3B, LCE1E, LCE3C, LCE1B, LCE2A, LCE1A, LCE2B, and LCE2C were associated with the patients' clinical stages (Figures 6E–H).

## Discussion

MMP25 is a member of the matrix metalloproteinase family which is frequently connected to embryonic development, reproduction, and tissue remodeling, as well as tumorigenesis. Although MMP25 has not been extensively investigated, research showed that MMP25 could regulate the chemotaxis of neutrophil and monocyte and generate “eat-me” signals to increase the phagocytic removal of neutrophils (17). MMP25 is highly expressed in human cancer cells, such as colon cancer cells and gastric cancer cells (18, 19). In addition, MMP25 was highly expressed and promoted tumor growth in colon cancer (18). Here, we report that the expression level of MMP25 correlated to the activated CD4^+^ memory T cells and prognosis in head and neck cancer. High expression levels of MMP25 were associated with a better survival outcome. Increased MMP25 expression level could impact the clinical stages of head and neck cancer patients, indicating that MMP25 expression could be used as a potential predictor of clinical stage and prognosis. Moreover, our analysis suggested that immune infiltration levels and diverse tumor-associated pathways were correlated with levels of MMP25 expression. Therefore, our study provides an insight into understanding the role of MMP25 in tumor immune environment and molecular mechanism in head and neck cancer.

In this research, we first analyzed the expression levels of MMPs and prognostic landscape in head and neck cancer. The differential expression between cancer and normal tissues was observed in many matrix metalloproteinase family genes. Expression levels of MMP10, MMP19, MMP24, and MMP25 were associated with prognosis in TCGA cohort of head and neck cancer by Kaplan-Meier analysis. Chongshan Wu demonstrated that the high expression of MMP19 was determined to be a poor prognostic factor in colorectal cancer (20). Trever G Bivona also suggested that MMP24 was a biomarker of tumor progression and worse outcomes in lung and/or gastric cancer patients (21). Univariate Cox proportional hazards regression demonstrated a significant correlation between MMP25 (HR=0.81, *p* = 0.04) and MMP8 (HR=1.26, *p* = 0.05) and prognosis of head and neck cancer patients. Notably, we observed that MMP25 was the only one gene that not only significantly expressed compared to the normal tissues but also played a significant role in the survival outcome of head and neck cancer patients by analysis of Kaplan–Meier method and univariate Cox proportional hazards regression. Likewise, recent evidence also revealed that high MMP25 expression levels were correlated with a better overall survival in ovarian cancer (22). Clinicopathological features from TCGA cohort of head and neck cancer showed that high levels of MMP25 expression were correlated with patients' clinical stages in our study. This important role of MMP25 has not been reported yet so far. Together, these findings suggest that high MMP25 expression levels may be used as a potential prognostic indicator in head and neck cancer.

Differential expressed genes analysis showed that the expression level of MMP25 was related to genes, such as IRF8, IKZF1, and DOCK2. Jason B. Muhitch et al. showed that high expression levels of IRF8 within metastatic sites prolonged overall survival of renal cell carcinoma compared to low levels of IRF8 expression (23). Overexpression of IKZF1 in melanomas enhanced the recruitment of immune infiltration and sensitivity to PD1 and CTLA4 inhibitors (24). Another study also showed that DOCK2 was significantly associated with survival outcome in colorectal cancer (25). In our study, the GO analysis of these differential expressed genes revealed that they were enriched in critical immune biological processes, including T cell activation, regulation of lymphocyte activation, immune response-regulating cell surface receptor signaling pathway, and so on. This partly explained the role of MMP25 in the activation of the CD4^+^ T memory cell by CIBERSORT analysis. Juric et al. revealed that MMP9 inhibition was able to promote T cell response by disruption of biochemical and physical barriers (26). The MMP23 expression of primary melanoma also demonstrated a trend toward an increased proportion of immunosuppressive Foxp3+ regulatory T cells. These results provided further support for the hypothesis that MMP25 could regulate the immune infiltration level due to the activation of downstream immunological molecules.

Furthermore, high expression levels of MMP25 were associated with many pathways in cancer, for instance, KRAS signaling pathway, apoptosis, PI3K/AKT/mTOR signaling pathway, and JAK/STAT signaling pathway. It has been revealed that JAK/STAT and mTOR pathways were significantly associated with poor overall survival (27, 28). Immune regulatory processes are largely driven by JAK-STAT signaling by a wide range of downstream cellular effectors, including oncogenes, miRNAs, DNA methylation, and other co-regulatory factors (29). Kozaki et al. found that PIK3CA mutations were relatively high in the late stage of oral cancer (30). Recent studies describe that the activation of KRAS signaling on cancer cells extends to the cancer microenvironment (31). Apoptosis is a mechanism that may contribute to cancer. Defects can occur at any point along apoptosis-associated pathways, leading to malignant transformation of the affected cells, tumor metastasis, and resistance (32). In addition, we identified key modules associated with head and neck cancer (HNSCC) patient's survival ratio and clinical stage based on the weighted gene co-expression network analysis (WGCNA). Hub genes have been identified, for example, APLP2, IFIT1, IFIT3, and CDC34. APLP2 expression was significantly related to disease-specific survival in renal cell carcinoma (33). It could be hypothesized that MMP25 was able to interact with multiple key genes to affect the progression of head and neck cancer. Taken together, it is possible that the regulation of cancer-associate genes and signaling pathways may be involved in the regulatory role of MMP25 in the clinical stages and prognosis in HNSCC.

Another important result of this study is that MMP25 expression correlated with diverse immune infiltration levels in head and neck cancer. Our results indicated that there was a correlation between MMP25 expression level and infiltration level of macrophages and B cells naïve, CD8^+^ T cells, activated CD4^+^ memory T cells, resting NK cells, M1 macrophage, and neutrophils, but lower M0 macrophage and eosinophil. Univariate Cox proportional hazards regression of these types of immune cells showed that activated CD4^+^ memory T cells correlated with lower hazard ratio (HR) for better overall survival (OS). Oberg showed that in patients with colon cancer, higher percentages of CD4^+^ memory T cells may be indicative of a better prognosis (34). Meanwhile, CD4^+^ memory T cells are associated with tumor cell metastasis to lymph nodes and tumor progression (35). Immune score was an algorithm providing scores of the immune infiltration level by ESTIMATE (13). In the present study, the high expression level of MMP25 showed a strong relationship with the immune scores between tumor tissues and normal tissues, as well as in different clinical stages. It is known that the immune scores could predict patients' clinical outcomes (36).

In summary, increased MMP25 expression correlates with better prognosis and increased immune infiltration levels in head and neck cancer, especially activated CD4+ memory T cells. Moreover, the expression of MMP25 potentially contributes to the regulation of tumor-associated pathway and oncogenes. Therefore, we propose that MMP25 probably plays an integral correlative role in immune cell infiltration and can be a potential prognosis biomarker in head and neck cancer. Further exploration of MMP25 function in clinical cohort study or *in vivo* and *in vitro* model will probably contribute to confirm these results.

## Data Availability Statement

Publicly available datasets were analyzed in this study. This data can be found here: https://portal.gdc.cancer.gov, https://cistrome.shinyapps.io/timer/.

## Author Contributions

YL and CG contributed to conception and design of the study and data analysis. KL, GZ, TW, and SZ contributed to data acquisition. GL and YL critically revised the manuscript. All authors contributed to the article and approved the submitted version.

## Conflict of Interest

The authors declare that the research was conducted in the absence of any commercial or financial relationships that could be construed as a potential conflict of interest.
