# Supplementary material for: MMP25 Regulates Immune Infiltration Level and Survival Outcome in Head and Neck Cancer Patients
Source: Front Oncol. 2020 Jul 29;10:1088. doi: 10.3389/fonc.2020.01088 (PMC7405909; doi:10.3389/fonc.2020.01088)
Supplement: Supplementary file 1 [file Table_1.docx]

**Supplemental Table 1**

KEGG analysis of genes in the significant module traits with the patients’ status of HNSC

| **Group** | **Clinical** | **Color** | **R** | ***p*-value** | **KEGG** |
| --- | --- | --- | --- | --- | --- |
| High | Status | lightsteelblue1 | 0.21 | 0.05 | hsa04060:Cytokine-cytokine receptor interaction |
|  |  | lavenderblush3 | 0.22 | 0.03 | hsa04610:Complement and coagulation cascades |
|  |  | greenyellow | −0.22 | 0.03 | hsa04510:Focal adhesion |
|  |  |  |  |  | hsa04512:ECM-receptor interaction |
|  |  |  |  |  | hsa05200:Pathways in cancer |
|  |  |  |  |  | hsa01100:Metabolic pathways |
|  |  |  |  |  | hsa04151:PI3K-Akt signaling pathway |
|  |  |  |  |  | hsa04110:Cell cycle |
|  |  |  |  |  | hsa04115:p53 signaling pathway |
|  |  |  |  |  | hsa04810:Regulation of actin cytoskeleton |
|  |  |  |  |  | hsa04390:Hippo signaling pathway |
|  |  |  |  |  | hsa04666:Fc gamma R-mediated phagocytosis |
| Low | Status | black | 0.26 | 0.01 | hsa04530:Tight junction |
|  |  |  |  |  | hsa04144:Endocytosis |
|  |  |  |  |  | hsa04014:Ras signaling pathway |
|  |  |  |  |  | hsa01212:Fatty acid metabolism |
|  |  |  |  |  | hsa04666:Fc gamma R-mediated phagocytosis |
|  |  |  |  |  | hsa04520:Adherens junction |
|  |  |  |  |  | hsa00071:Fatty acid degradation |
|  |  |  |  |  | hsa04146:Peroxisome |
|  |  |  |  |  | hsa05231:Choline metabolism in cancer |
|  |  | darkslateblue | −0.2 | 0.04 | hsa04014:Ras signaling pathway |
|  |  |  |  |  | hsa04010:MAPK signaling pathway |
|  |  | darkorange2 | −0.31 | 0.001 | hsa04120:Ubiquitin mediated proteolysis |
|  |  |  |  |  | hsa05230:Central carbon metabolism in cancer |
|  |  | darkseagreen4 | 0.2 | 0.04 | hsa04144:Endocytosis |
|  |  |  |  |  | hsa04062:Chemokine signaling pathway |
|  |  |  |  |  | hsa04014:Ras signaling pathway |
|  |  |  |  |  | hsa04010:MAPK signaling pathway |
