# Supplementary material for: MMP25 Regulates Immune Infiltration Level and Survival Outcome in Head and Neck Cancer Patients
Source: Front Oncol. 2020 Jul 29;10:1088. doi: 10.3389/fonc.2020.01088 (PMC7405909; doi:10.3389/fonc.2020.01088)
Supplement: Supplementary file 2 [file Table_2.docx]

**Supplemental Table 2**

KEGG analysis of genes in the significant module traits with the clinical stage of head and neck cancer

| **Group** | **Clinical** | **Color** | **R** | ***p* value** | **KEGG** |
| --- | --- | --- | --- | --- | --- |
| High | Stage | palevioletred3 | −0.26 | 0.01 | hsa04141:Protein processing in endoplasmic reticulum |
|  |  | tan | −0.22 | 0.04 | hsa04612:Antigen processing and presentation |
|  |  |  |  |  | hsa04514:Cell adhesion molecules (CAMs) |
|  |  |  |  |  | hsa04623:Cytosolic DNA-sensing pathway |
|  |  |  |  |  | hsa04668:TNF signaling pathway |
|  |  |  |  |  | hsa04622:RIG-I-like receptor signaling pathway |
|  |  |  |  |  | hsa04060:Cytokine-cytokine receptor interaction |
|  |  |  |  |  | hsa04620:Toll-like receptor signaling pathway |
|  |  |  |  |  | hsa04210:Apoptosis |
|  |  |  |  |  | hsa05203:Viral carcinogenesis |
| Low | Stage | honeydew1 | −0.25 | 0.01 | hsa00510:N-Glycan biosynthesis |
|  |  |  |  |  | hsa00020:Citrate cycle (TCA cycle) |
|  |  | navajowhite2 | −0.22 | 0.03 | hsa00600:Sphingolipid metabolism |
|  |  | salmon | −0.25 | 0.01 | hsa04514:Cell adhesion molecules (CAMs) |
|  |  |  |  |  | hsa04022:cGMP-PKG signaling pathway |
|  |  | darkorange | −0.27 | 0.006 | hsa00531:Glycosaminoglycan degradation |
|  |  | tan | 0.21 | 0.04 | hsa03030:DNA replication |
|  |  |  |  |  | hsa04110:Cell cycle |
|  |  |  |  |  | hsa03440:Homologous recombination |
|  |  |  |  |  | hsa03430:Mismatch repair |
|  |  |  |  |  | hsa03420:Nucleotide excision repair |
|  |  |  |  |  | hsa03410:Base excision repair |
|  |  |  |  |  | hsa04115:p53 signaling pathway |
|  |  | darkturquoise | −0.22 | 0.02 | hsa04510:Focal adhesion |
|  |  |  |  |  | hsa04350:TGF-beta signaling pathway |
|  |  |  |  |  | hsa04512:ECM-receptor interaction |
|  |  |  |  |  | hsa04060:Cytokine-cytokine receptor interaction |
|  |  |  |  |  | hsa04668:TNF signaling pathway |
|  |  |  |  |  | hsa04621:NOD-like receptor signaling pathway |
|  |  |  |  |  | hsa04151:PI3K-Akt signaling pathway |
