# Supplementary material for: MMP25 Regulates Immune Infiltration Level and Survival Outcome in Head and Neck Cancer Patients
Source: Front Oncol. 2020 Jul 29;10:1088. doi: 10.3389/fonc.2020.01088 (PMC7405909; doi:10.3389/fonc.2020.01088)
Supplement: Supplementary file 3 [file Data_Sheet_1.docx]

Supplemental Figure 1


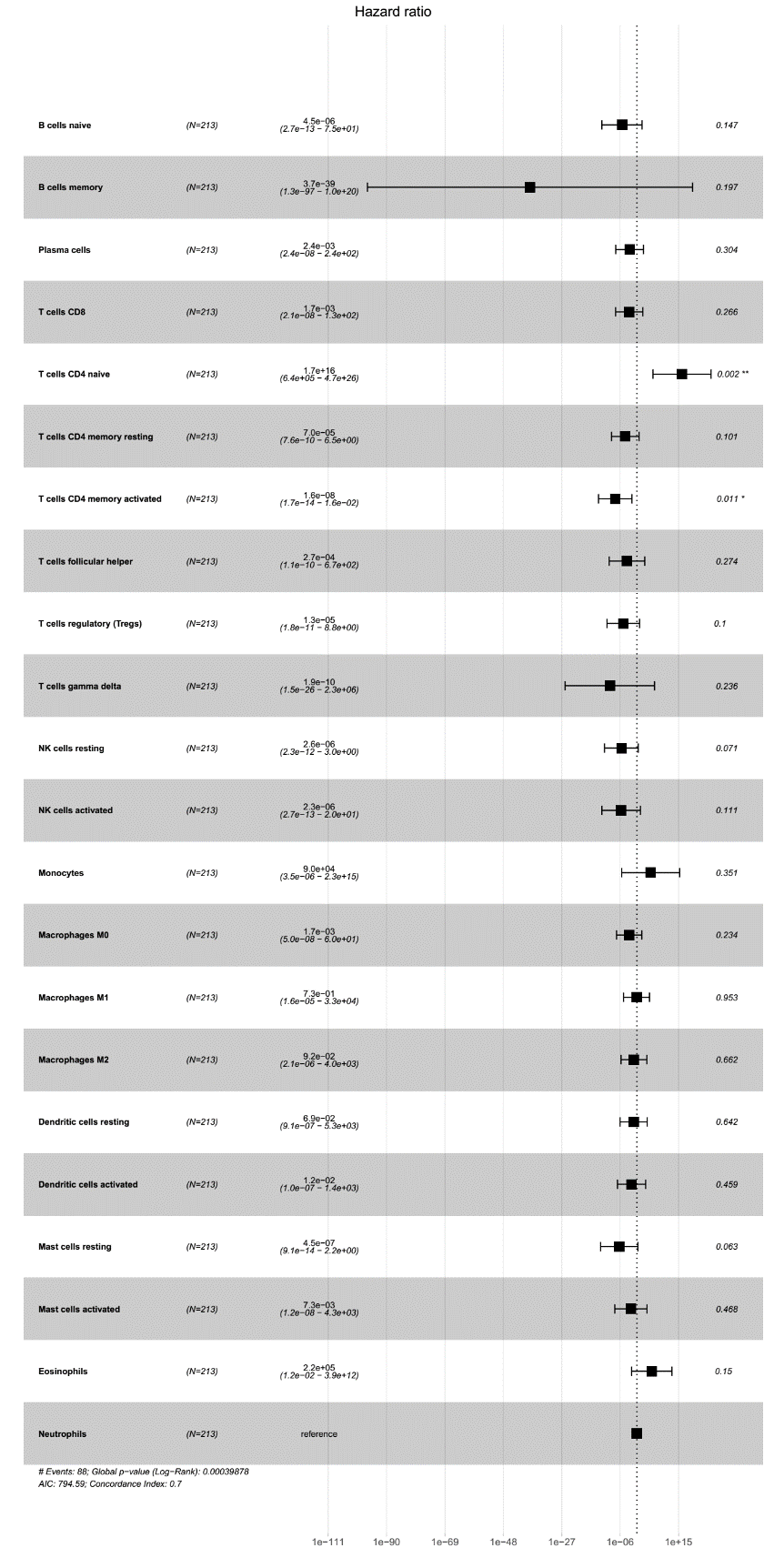


A

**Figure S1** The forest plot exhibited hazard ratios (HR) and 95% confidence interval (95%CI) by multivariate Cox regression analysis of immune infiltration.
