# Supplementary material for: MMP25 Regulates Immune Infiltration Level and Survival Outcome in Head and Neck Cancer Patients
Source: Front Oncol. 2020 Jul 29;10:1088. doi: 10.3389/fonc.2020.01088 (PMC7405909; doi:10.3389/fonc.2020.01088)
Supplement: Supplementary file 4 [file Data_Sheet_2.docx]

**Supplemental Figure 2**


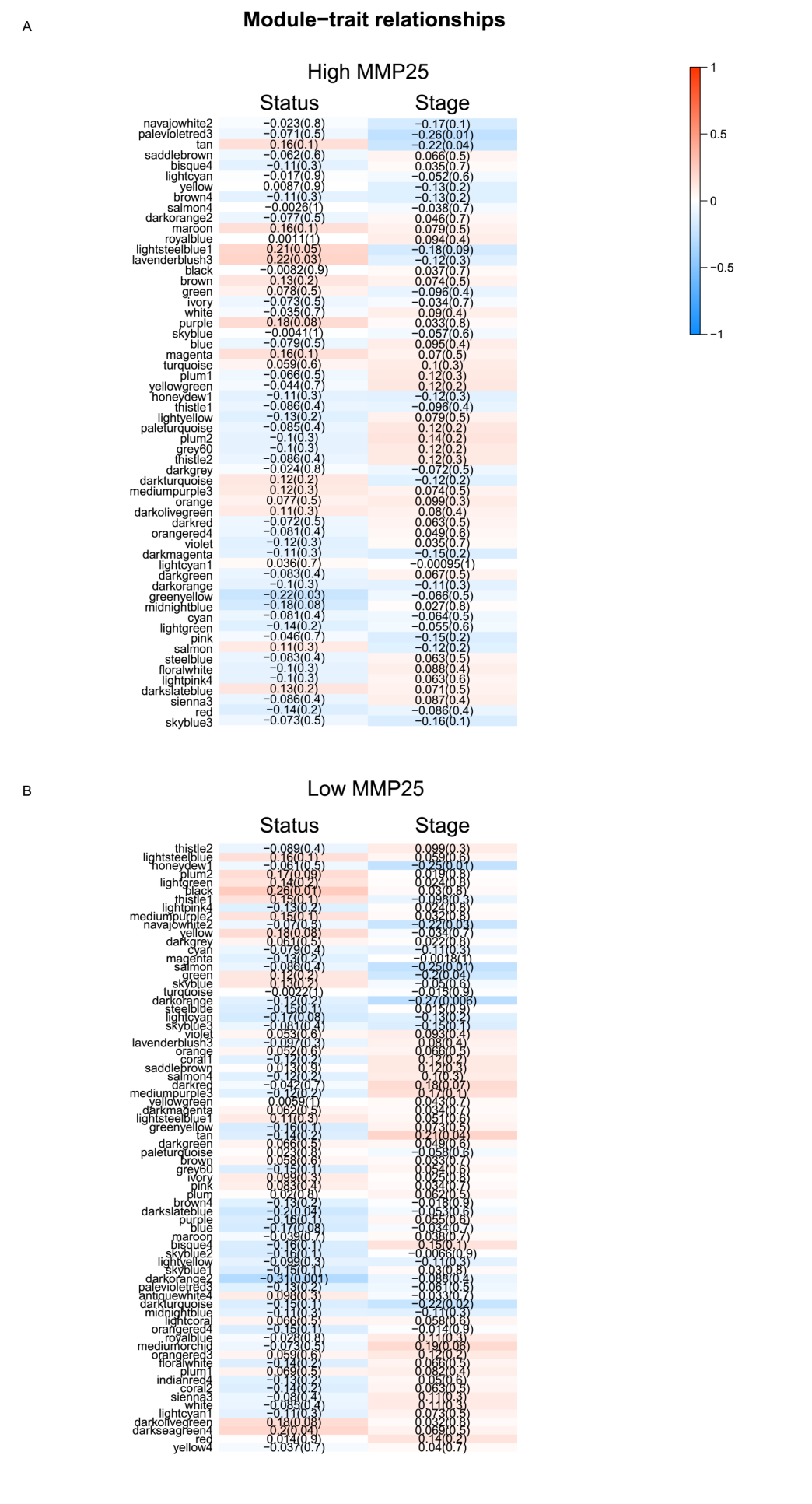


**Figure S2** Heatmap of the correlation between module eigengenes and clinical traits (A) Module-trait relationships of the MMP25 high expression group. (B) Module-trait relationships of the MMP25 low expression group. Each gene module was shown to exhibit the correlation and p-value with the patients’ status and clinical stage.
